# Bilateral one-stage single-port sympathicotomy in primary focal hyperhidrosis, a prospective cohort study: treat earlier?

**DOI:** 10.1186/s13019-021-01430-0

**Published:** 2021-03-25

**Authors:** Michiel Kuijpers, Gwen Peeters, Petra W. Harms, Wobbe Bouma, Mike J. DeJongste, Massimo A. Mariani, Theo J. Klinkenberg

**Affiliations:** 1 Department of Cardiothoracic Surgery, University Medical Center Groningen, AB32, Postbus 30.001, Groningen, 9700 RB The Netherlands; 2 Hyperhidrosis Expert Center, Dermatology, Martini Hospital, Groningen, The Netherlands; 3 University Medical Center Groningen, Groningen, The Netherlands

**Keywords:** Palmar, Hyperhidrosis, Sweating, Sympathicotomy, Sympathectomy, VATS, Single-port

## Abstract

**Background:**

Primary Focal Hyperhidrosis (PFH) has a detrimental effect on Quality of Life. Repetitive, non-curative symptomatic strategies dominate current treatment of PFH, in spite of the availability of an effective and permanent curative treatment like Endoscopic Thoracic Sympathectomy (ETS). Current surgical optimization may allow for a re-established position of sympathetic modulation in this treatment algorithm. We sought to evaluate the safety, effectiveness, and long-term results of a Bilateral One-stage Single-port Sympathicotomy (BOSS) procedure in PFH patients and to identify subgroups benefitting most.

**Methods:**

Prospective analysis of 163 patients, 35 (21.5%) underwent Rib-3 (R3) BOSS for palmar PFH, 58 (35.6%) R3-R5 BOSS for axillary PFH and 70 (42.9%) R3-R5 BOSS for combined palmar/axillary PFH. Effectiveness was measured using Skindex-29 and the Hyperhidrosis Disease Severity Scale (HDSS).

**Results:**

Overall Skindex-29-rating (46.5 ± 14.8 preoperatively vs 20.1 ± 20.6 postoperatively, *p* < 0.001), and HDSS score (3.71 ± 0.45 preoperatively vs 1.82 ± 0.86 postoperatively, *p* < 0.001) indicated a significant improvement in health-related quality of life after BOSS. R3 BOSS was superior to R3-R5 BOSS in terms of HDSS score (1.49 vs 1.91 respectively, *p* = 0.004) and in terms of severe compensatory hyperhidrosis, a frequently reported side-effect (17.1% vs 32.8% respectively, *p* < 0.001). No major complications occurred.

**Conclusions:**

BOSS is safe, effective, and offers a long-term curative solution in the treatment of PFH. Especially in the palmar PFH subgroup, R3 BOSS treatment results compare favorably to the treatment results of non-curative alternatives published in the current literature. Therefore, R3 BOSS should be offered to all patients with severe PFH, reporting insufficient benefit of treatment options such as oral and/or local agents.

## Background

Hyperhidrosis is a pathologic condition in which sweat production exceeds the need for physiological thermoregulation. It may develop secondary to psychological causes or a variety of medical disorders, but is mostly cryptogenic / primary, thus without an identifiable underlying medical disorder. Primary Focal Hyperhidrosis (PFH) is usually severe and symmetrical with a focus on the palms, axillae, and feet. PFH, and its associated symptoms have a major impact on Health-Related Quality of Life (HRQL) [1]. An increasing prevalence is seen over time, ranging from 0.6% in the past to almost 5% in recent literature [2]. Current treatment options consist of 1) topical solutions like aluminum chloride, 2) intradermal application of sweat gland blockers like botulinum toxin, 3) oral anticholinergics including oxybutynin and glycopyrrolate, 4) iontophoresis and 5) Endoscopic Thoracic Sympathectomy (ETS). The first four options offer temporary result; discontinuation of these treatments is followed by certain relapse, since they are fundamentally non-curative in nature [3, 4].

Repeating these non-curative symptomatic strategies seems futile, as ETS, a permanent curative treatment without systemic side-effects, exists. ETS is currently only offered to patients at the end of the treatment algorithm or even not at all due to three arguments: 1) unfamiliarity of medical professionals with the ‘modern-day’ ETS procedure, 2) prejudice and unjust perceived surgical invasiveness, and 3) fear to induce Compensatory Hyperhidrosis (CH) i.e. new postoperative hyperhidrosis in a non-targeted area [5, 6].

Thoracic sympathetic surgery is not a novel treatment. In 1934 Leriche was the first to perform a sympathectomy for PFH [7], although surgical access has dramatically improved since the introduction of Video-Assisted Thoracic Surgery (VATS). While the first thoracoscopic technique was described in the earlier 1950’s by Kux, a series of 55 patients who had excellent results after thoracoscopic sympathectomy (TS) for hyperhidrosis [8], large series of conventional multiportal endoscopic thoracic sympathectomy were performed in the late nineties of the twentieth century reporting improved quality of life and treatment satisfaction of 93–95% with success rates of 71–100% [9, 10]. Recent further optimization of technique led to standardized and reproducible single-port techniques, allowing for bilateral treatment in a single session while minimizing post-operative discomfort [11, 12].

Historically ETS consisted of a true sympathectomy by removing a segment of the sympathetic nerve including multiple ganglia. More recently the switch to a sympathicotomy was made, with transection of the sympathetic nerve while striving to save the ganglia [13]. It is hypothesized that reducing damage to the ganglia may reduce CH severity by leaving crucial feedback-loops intact [14–16]. Furthermore, surgical optimization resulted in a minimally invasive uniportal one-stage bilateral technique [12]. Given these improvements, aforementioned arguments 2 and 3 are waived, and argument 1 should be addressed by raising awareness.

### Study objective

To evaluate the long-term results, safety and effectiveness of a Bilateral One-stage Single-port Sympathicotomy (BOSS) in patients suffering from PFH and to identify which patient groups benefit most from the procedure.

## Methods

### Baseline characteristics

This study was conducted with the formal and written approval of the Dutch Medical Ethics Comity (METc UMCG 2015/104). A prospective cohort of 172 patients suffering from PFH received BOSS. Follow-up after 18 months (82.5 ± 8.2 weeks) was complete in 163 patients (94.8%), reflecting 326 procedures, by returning a response form and questionnaire. Nine patients were lost to follow-up. Eighty-six patients were female (52.8%) and 77 were male (47.2%), with a mean age of 30.9 ± 9.8 years and a mean Body Mass Index (BMI) of 24.0 ± 3.6 kg/m^2^. Thirty-five patients (21.5%) underwent R3 BOSS for palmar PFH, 58 (35.6%) R3-R5 BOSS for axillary PFH and 70 (42.9%) R3-R5 BOSS for combined palmar/axillary PFH. Pre-operative patient data are presented in Table 1.

**Table 1:** Preoperative patient data (*n* = 163)

Variable ^a^	Value
Age (years)	30.9 ± 9.8
Male	77 (47.2)
Female	86 (52.8)
BMI (kg/m^2^)	24.0 ± 3.6
Form of PFH	
Isolated Palmar	35 (21.5)
Isolated Axillary	58 (35.6)
Combined Palmar/Axillary	70 (42.9)
HDSS Classification	
Score 1 and 2 (Mild/Moderate)	0 (0)
Score 3 (Severe)	46 (28.2)
Score 4 (Intolerable)	117 (71.8)

^a^ Data are presented as mean ± standard deviation or number (%)

*BMI* Body Mass Index, *PFH* Primary Focal Hyperhidrosis, *HDSS* Hyperhidrosis Disease Severity Scale

We defined PFH according to the diagnostic criteria described by Hornberger et al [17]. The Skindex-29 questionnaire is a validated HRQL measure of skin diseases and consists of 30 questions related to the impact of PFH on everyday HRQL. Twenty-nine questions (except question 18, that analyses a side-effect of treatment) are assigned to three domain scores, separating impact on emotions, functioning and symptoms [18]. Scores are expressed on a 100-point scale, with higher scores indicating lower levels of quality of life. The clinically important cut-off scores for severely impaired HRQL are ≥39 on emotions, ≥ 37 on functioning, ≥52 on symptoms, and ≥ 44 on the overall score [19].

**Fig. 1:**
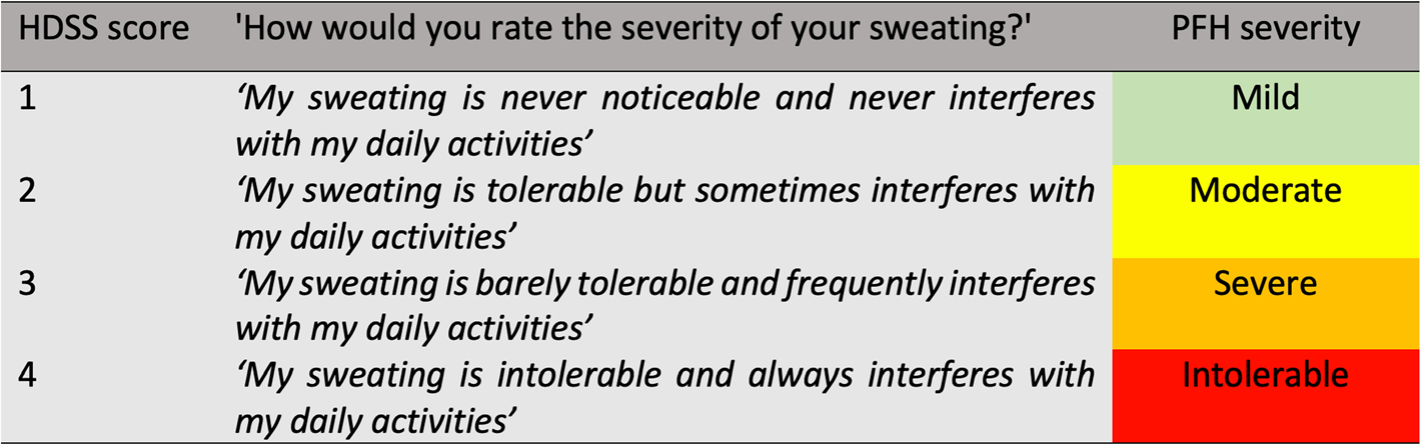
Hyperhidrosis Disease Severity Scale (HDSS). Patients scoring a 3 or 4 qualified for surgery

Severity of PFH was classified according to the 4-point Hyperhidrosis Disease Severity Scale (HDSS), a disease-specific scale for PFH patients providing a qualitative measure based on the detrimental effect of hyperhidrosis on daily activities (Fig. 1). Patients with a HDSS score of ≥3, classifying hyperhidrosis as severe or intolerable, qualified for surgery. A post-operative 1-point improvement in HDSS score is associated with a 50% reduction in sweat production; a post-operative 2-point improvement constitutes an 80% reduction [6]. Informed consent was obtained from all patients.

### Surgical protocol

**Fig. 2:**
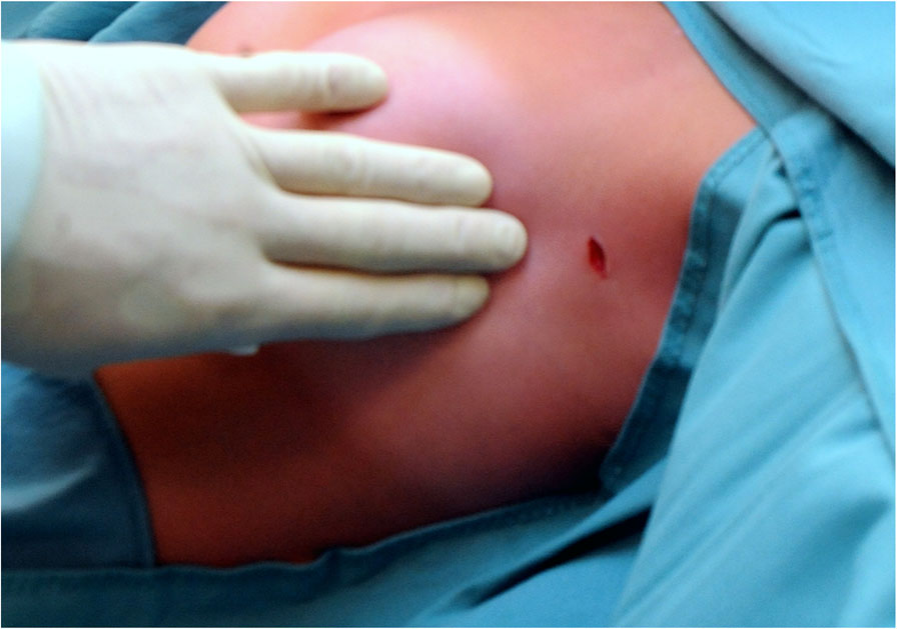
Single port trans axillary VATS. A single 7 mm incision for single port-insertion in the left anterior axillary line at the level of the thirds intercostal space

Our surgical technique has been previously described [12]. In short: patients were seated at a 45° angle, which improves the intra-thoracic field of view by forcing the collapsed lung dorso-caudally and which allows a one-stage bilateral procedure. General anesthesia was administered. Initially single lung ventilation was obtained using a double lumen endotracheal tube or single lumen endotracheal tube with unilateral blocker (EZ-blocker Inc., Delft, The Netherlands). After 54 procedures we switched to a single lumen endotracheal tube and performed BOSS under apnea, further simplifying the procedure. The incision area was infiltrated with 5 ml of bupivacaine 0,25% from skin to costal periosteum to reduce direct postoperative pain. A 7 mm incision was made in the anterior axillary line at the level of the third intercostal space. Under apnea, a 5 mm straight scope (Olympus Medical System, Tokyo, Japan) was inserted through a 7 mm trocar (Fig. 2).

CO2 insufflation was initiated when collapse of the lung was insufficient. The trocar was retrieved allowing the cautery hook to be introduced. The sympathetic chain was identified including the first, second and third rib. The sympathetic chain was then divided using electro cautery on the surface of the third rib (R3) or third, fourth and fifth rib (R3-R5). This transection was extended 2 cm laterally over the rib to transect accessory nerve fibers in all cases. A thoracic drain was inserted and under visual control the lung was re-insufflated followed by 2-min recruitment. After 2-min recruitment the thoracic drain was removed under positive end-expiratory pressure of 30 cm H_2_O. The skin was closed with an intracutaneous suture. The surgical procedures were conducted first on the right side followed by the left side in an identical manner. Operation time, level of sympathicotomy and duration of hospital stay were recorded.

### Follow-up

The primary end point was improvement in palmar, axillary, or axillary/palmar PFH as measured by HDSS scores pre-operatively, 2 weeks post-operatively, 18 months postoperatively and by comparing Skindex-29 questionnaires taken pre-operatively and at 18 months follow-up. Secondary end points were overall procedural satisfaction, peri-operative complications, post-operative pain requiring analgesics and presence of CH. Using a 5-point satisfaction scale, participants had to indicate their satisfaction with the achieved result by choosing one of the following: very satisfied (=5), satisfied (=4), not satisfied nor dissatisfied (=3), reasonably satisfied (=2) or dissatisfied (=1). Potential peri-operative complications, such as bleeding, pneumothorax, Horner syndrome and wound infections were recorded during follow-up. Location and extent of CH was evaluated by asking the patient specifically about the occurrence of increased sweating in body areas that did not exhibit excess sweating, preoperatively.

### Statistical analysis

Statistical analysis was performed using SPSS 21.0 software (IBM Corp, Armonk, NY). Continuous variables were expressed as mean ± SD or median and normal range. Categorical variables were expressed as percentages. The paired samples t-test was used for comparing preoperative and postoperative HDSS score. Data were displayed in a box-and-whisker plot. A *p*-value less than 0.05 was considered significant.

## Results

Off 172 patients, nine were lost to follow-up at 18 months. All lost patients reported a HDSS score of 1 at two-week follow-up, and there were no complications during or after surgery within the first 2 weeks in this group. Off the 163 patients (94.8%) included in 18 months follow-up, the mean operative time was 40.5 ± 17.2 min. There was no significant difference in operating time between R3 or R3-R5 BOSS. The average hospital stay was 1.06 ± 0.36 days, meaning that 95.7% of the patients were discharged the day after the procedure. No major complications such as bleeding, conversion to thoracotomy and Horner’s syndrome occurred. Residual pneumothorax occurred in six procedures (1.8%), requiring drainage using a pleural catheter. Of these six patients, five were discharged the day after the procedure and one was discharged at day five postoperatively. All patients fully recovered. The mean duration of postoperative pain, defined as requiring any form of analgesics, was 4.8 ± 3.2 days.

### Improvement in PFH

Patients with palmar, axillary, or combined palmar/axillary PFH had the same average preoperative HDSS score of 3.71 ± 0.45. With the effect of the operation being immediate, the HDSS score declined significantly to a mean of 1.04 ± 0.28 at 2 weeks follow-up. At 18 months follow-up, mean HDSS was 1.82 ± 0.86, (*p* < 0.0001) revealing a lasting operative result. Almost all patients (99.4%) reported a ≥ 2-point reduction in HDSS score 2 weeks after the procedure. After 18 months of follow-up, 112 patients (68.7%) still reported a ≥ 2-point reduction in HDSS score and 37 (22.7%) patients had still a 1-point reduction in HDSS score. In 13 patients (8.0%) the HDSS score was not affected by the intervention after 18 months. One patient (0.6%) remained in HDSS class 4, due to severe CH in the groins.

### Quality of life, compensatory hyperhidrosis and patient satisfaction

At 18 months follow-up the overall mean Skindex-29-rating decreased from 46.5 ± 14.8 to 20.1 ± 20.6, indicating a significant improvement in HRQL (*p* < 0.01). CH, occurring at any moment after surgery, was rated as none to moderate (HDSS 0–2) in 115 patients (70.6%) and severe (HDSS 3–4) in 48 patients (29.4%). Most affected body areas were the back (41.9%), abdominal region (14.5%) and breast region (13.4%). No relationship between severity of the CH and the affected body areas could be identified. No other side-effects were reported. At 18 months follow-up, 87 patients (53.4%) were very satisfied and 52 patients (31,9%) were satisfied. Twenty-four patients (14.7%) were not satisfied with the obtained result; in 16 patients (9.8%) this was due to the fact that the obtained results did not meet their expectations, and the remaining 8 patients (4.9%) were unsatisfied as a result of severe CH. Overall recommendation rate (‘Would you recommend this procedure, considering the obtained effect’) was excellent at 87.7%.

At 18 months follow-up the palmar hyperhidrosis group receiving isolated R3 BOSS scored significantly higher on all measured domains than the combined palmar/axillary or isolated axillary group receiving R3-R5 BOSS. Satisfaction scores (4.49 vs 3.95, *p* = 0.028), satisfaction rate (94.3% vs 82.8%, *p* = 0.003), post-operative HDSS score (1.49 vs 1.91, *p* = 0.004), severe CH-rate (17.1% vs 32.8% *p* < 0.001), and recommendation rate (94.3% vs 85.2% *p* = 0.002) proved to be superior in the R3 BOSS cohort in comparison with the R3-R5 BOSS cohort. Impact on HRQL did not significantly differ between those two groups (Table 2).

**Table 2:** R3 BOSS vs. R3-R5 BOSS at long term follow-up (*n* = 163)

Variable ^a^	R3-BOSS *n* = 35	R3-R5 BOSS *n* = 128	*p*-value
HDSS-score			
Baseline	3.72 ± 0.45	3.71 ± 0.46	0.959
LTFU	1.47 ± 0.70	1.91 ± 0.88	0.004
Skindex-29 Overall score			
Baseline	41.1 ± 15.3	48.0 ± 14.4	0.014
LTFU	14.3 ± 20.0	21.7 ± 20.5	0.060
Severe CH-rating	17.1	32.8	< 0.001
Recommendation rating	94.3%	85.2%	0.002
Satisfaction rate (1–5)	4.49	3.95	0.028
Satisfied with obtained result	94.3%	82.8%	0.003

^a^ Data are presented as mean ± standard deviation or number (%). *HDSS* Hyperhidrosis Disease Severity Scale, *CH* Compensatory Hyperhidrosis, *LTFU* Long Term Follow Up

## Discussion

PFH is a highly underestimated medical problem with huge social impact and detrimental effect on quality of life. In fact, when the same scale is used to measure the intrusive impact on HRQL, it is rated higher by PFH patients than by patients suffering from end-stage renal disease, multiple sclerosis or rheumatoid arthritis [20]. Understandably, an effective, reliable and definitive treatment is desired by an increasing number of patients [1–4]. The pathogenesis of PFH is relatively unclear, hindering causal treatment. In general, symptomatic dermal or subcutaneous treatment options are offered to hinder or block sweat glands, which lowers sweat production locally. These treatments include, but are not limited to, iontophoresis and botulinum toxin injections. Most patients have a non-lasting treatment response on the aforementioned treatments and satisfaction rates are low due to side-effects, adverse events, and/or inadequate response [21].

The sudomotor innervation of the sweat glands is provided through the cholinergic sympathetic nervous system. In patients suffering from PFH choline acetyltransferase and vasoactive intestinal peptide, measured in sympathetic ganglia, proved to be significantly increased compared to individuals not suffering from PFH, making PFH the result of sympathetic overstimulation [22]. Addressing this sympathetic overstimulation by systemic anticholinergics is a potential successful treatment for PFH but is mainly limited by its side effects. Most data are available for oral glycopyrrolate and oxybutynin treatment. Improvement rates for oral glycopyrrolate range from 67 to 90% in two retrospective studies, in which 13.3% of patients were listed as non-responder and 20% of the patients discontinued treatment due to side effects [23, 24]. A randomized placebo-controlled trial for oxybutynin in general hyperhidrosis showed a 60% improvement in HDSS, with 43% of patients reporting a dry mouth as a side effect [25]. While cost-effectiveness and the non-invasive nature of oral agents are obviously advantageous, most studies on oral medical treatment for PFH are limited in their follow-up and lack of information about long-term efficacy and safety. Therefore, thoracic sympathetic surgery, which modifies the sympathetic signals halfway down the tract, seems to be an appealing alternative. If one is willing to accept general anesthesia, a 24-h hospital stay and the possible negative effect postoperative pain can have on the days following the procedure, it offers a safe and long-term effective treatment alternative.

Instead of true sympathectomy (surgically removing a part of the sympathetic chain including one or more ganglia), a sympathicotomy, in which the ganglia are left unaffected is now performed. Although the pathogenic mechanism of CH is poorly understood, it is hypothesized that severing sympathetic reflex arcs that run through the ganglia to the hypothalamus, lead to CH through dysfunctional sweat regulation in the affected body parts [13]. This finding corroborates with earlier studies, showing higher rates of dissatisfaction and severe CH in patients treated for axillary PFH when compared to patients treated for palmar PFH [26], and so adds to the understanding that the ganglia should be left untouched.

CH remains the only lasting cause for discontent postoperatively. It is of interest to note that CH is however still preferred by most patients over the distress experienced from PFH. Only 8 out of the 48 patients (17%) experiencing severe CH at any moment after surgery were not satisfied with the obtained result. It must be stressed that CH is a side-effect of surgery on the sympathetic nerve and not a complication of the surgery. The key for accepting and living with CH after BOSS lies in thorough and accurate pre-operative information about its risk [17]. The importance of thorough pre-operative counseling and consent cannot be overstated.

### Subgroups

The main objective of the study was to identify which subgroup(s) benefitted most from the procedure. In the long-term, HDSS score reduction, and satisfaction with surgery are better maintained in the R3 than in the R3–5 BOSS group. We found that isolated R3 BOSS offers the best compromise between treating PFH and the risk of CH (Table 2). In other words: a more extensive sympathetic denervation is correlated to a significant higher long-term incidence of CH.

Although not subject of the present study, the optimal level of sympathicotomy, balancing between obtained effect and CH risk reduction, is frequently debated in literature. While our choice for a R3 level sympathicotomy for isolated palmar PFH is supported by studies reporting sympthicotomy at R3 to be more effective than at R2 [27], a more recent systematic review suggests R4-sympathicotomy to be superior to isolated R2 or R3 sympathicotomy ([28]. For combined palmar/axillary or isolated axillary PFH, the CH rate may be beneficial affected by reducing the levels of intervention, while efficacy is maintained [29]. The common denominator, irrespective of indication, seems to be that if R2 is not ‘touched’, less CH is observed [30, 31].

While R3 BOSS for isolated palmar PFH proved most effective in our cohort, it also compares favorably with the alternatives. Oxybutynin for treatment of palmar PFH was effective in 80% of patients and HRQL improved in 74.6% of patients [32]. Botulinum toxin proved to be a more effective treatment modality for palmar hyperhidrosis than iontophoresis with aluminum chloride (80% vs 47%, *p* = 0.007) [33]. The persistence of improvement in HRQL for botulinum toxin therapy was on average only 4 months with frequent serious side-effects such as disturbed neuromuscular transmission and generalized neurological symptoms [34].

Botulinum toxin showed to be inferior to ETS in the treatment of palmar hyperhidrosis, with a significant reduction in the ETS-group: 94% versus 63% at 6 months (*p* = 0.036) and 94% versus 30% at 12 months (*p* = 0.011). Patient’s satisfaction after 6 months (*p* = 0.04) and 1 year (*p* = 0.001) was significantly higher in the ETS group [35]. Our study adds to a growing body of evidence, which shows that a minimally invasive sympathicotomy is superior to non-surgical treatment options for palmar hyperhidrosis.

Two limitations of this study merit attention. Firstly, data presented are the data as reported by the patient when returning the response form to MK. Data therefore is not validated by an independent investigator. Secondly, there might be bias in our cohort since arguably the more severe cases of PFH were referred for sympathicotomy, overestimating the positive results in our study.

## Conclusion

In conclusion, Bilateral One-stage Single-port Sympathicotomy is, in experienced hands, a safe and effective procedure in the treatment of PFH. It offers a definite relief of complaints of PFH and compares favorably to the existing literature on alternatives as medication, botulinum toxin and iontophoresis in the palmar subgroup. Re-establishing the position of modern-day ETS in the treatment algorithm of palmar PFH is called for. We suggest that R3 BOSS should be offered to all patients with severe palmar PFH reporting insufficient benefit of alternative treatment options. Thorough pre-operative counseling is key in determining postoperative satisfaction.

## Data Availability

The dataset used and/or analysed during the current study are available from the corresponding author on reasonable request.

## Abbreviations

BMI: Body Mass Index
BOSS: Bilateral One-stage Single-port Sympathicotomy
CH: Compensatory Hyperhidrosis
ETS: Endoscopic Thoracic Sympathectomy
HRQL: Health-Related Quality of Life
HDSS: Hyperhidrosis Disease Severity Scale
LTFU: Long Term Follow Up
PFH: Primary Focal Hyperhidrosis
VATS: Video Assisted Thoracic Surgery
